# Children's Eyesight and the Cinematograph

**Published:** 1917-03-03

**Authors:** 


### CHILDREN'S EYESIGHT AND THE CINEMATOGRAPH.

New pleasures bring with them new penalties, and the cinematograph, it appears, comes within the scope of this doctrine. Many people have discovered the truth for themselves, and Mr. Bishop Harman, in a recent paper in the *British Medical Journal*, has analysed the evil and has set himself to devise an appropriate remedy. His concern is not with the moral ills which result from a contemplation of the exploits of triumphant villains or of dare-devil heroines, but with the physical consequences which the lantern-show may produce on the eyesight of its youthful admirers. Happily these consequences are not of a very serious order. There is "glare," there is "flicker," there is "rapidity of movement," there is "concentration." Yet the evidences of actual harm hardly reach the anticipations aroused by this formidable catalogue. All the various factors are set out with scientific precision, and the statement engenders the conviction that so terrible a series of hostile agencies must inevitably produce widespread suffering and disaster.

Yet the proofs of such misfortunes are sadly to seek. It seems that the records of various eye-clinics show an increasing number of children who, though they have failed to pass the standard vision tests at school, are found by the surgeons to have normal, or approximately normal, eyes. The youngsters, it is allowed, may fail from various causes, but as "a condition of fatigue" is one of the causes, and as Mr. Harman has ascertained that "in some cases" children belonging to the group here in question are in the habit of going to picture-shows, the cinematograph is suggested as the responsible agent. And, still further to heighten the indictment, if children with normal eyes are thus affected, it is certain, we are told, that more serious evils must fall on those with defective vision. On this count, however, the prosecutor offers no evidence.

The case, as thus presented, is certainly a very weak one, and might, without any failure of courtesy, be classed as an example of special pleading. The expert, in his fulness of knowledge, tells us that certain influences must do harm, and we hear his recital with becoming awe. So long as he remains on his pedestal we continue to tremble, even though our own sensations assure us that we are still fairly happy. But when he descends to evidence, we know something of the rules of the game. His propositions now are those of an ordinary mortal, and we examine them free from the terrors of authority. This is the mistake, if we may venture to say so, that Mr. Harman has made. Had he merely told us of the scientific wickedness of "flicker," and of "glare," and the rest, we should have been greatly impressed and have been ready to place the picture-palace on the index. But as he has elected to rely on the evidence of experience, and as this amounts to little more than an announcement that "some" of the children he has examined were *habitués* of the cinematograph exhibitions, we really are not able to give him a favourable verdict. It is the natural ambition of the expert to make our flesh creep, and in the pursuit of this aim he is in danger of overstating his case. We give him a most respectful hearing, but if he will insist not on authority but on argument he must submit to the usual standards. To "open" a stronger case than the evidence will support is not usually a successful forensic expedient. Indeed, a good case may be spoiled by it.

